# Multiple Myeloma Side Population Cells Promote Dexamethasone Resistance of Main Population Cells through Exosome Metastasis of LncRNA SNHG16

**DOI:** 10.1155/2023/5135445

**Published:** 2023-02-09

**Authors:** Xi Yang, Zenghua Lin, Haiyan Liu, Xinfeng Wang, Hong Liu

**Affiliations:** Department of Hematology, Affiliated Hospital of NanTong University, Nantong 226000, China

## Abstract

**Background:**

The emergence of dexamethasone (Dex) resistance limits its efficacy. Side population (SP) cells in MM have strong tumorigenicity. Nevertheless, the detailed effect by which SP cells regulate Dex resistance in MP cells has not been completely verified and needs to be further investigated.

**Methods:**

SP and MP cells were sorted from RPMI-8226. mRNA expression and cell viability were analyzed using quantitative real-time PCR (qRT-PCR) and MTS assays, respectively. The presence of exosomal lncRNA SNHG16 was verified by transmission electron microscopy, differential ultracentrifugation, and qRT-PCR. Protein expression levels were measured using western blotting. Gain or loss function analyses were performed to demonstrate the role of SNHG16 in the Dex resistance of MP cells.

**Results:**

Dex resistance of SP cells was remarkably stronger than that of MP cells. Compared with MP cells, the survival rate and Dex resistance of MP cells cotreated with SP cell-derived exosomes were increased. SNHG16 expression was significantly enhanced in SP cell-derived exosomes compared to MP cell-derived exosomes. SNHG16 expression was remarkably increased in MP cells transfected with OE-SNHG16 vectors, and Dex resistance of MP cells was enhanced. When SNHG16 was silenced in SP cells, the SNHG16 expression was downregulated in both SP cells and SP cell-derived exosomes. SNHG16 expression and Dex resistance were both remarkably downregulated in MP cells treated with SP-si-SNHG16-exosomes compared to MP cells treated with SP-si-NC-exosomes.

**Conclusion:**

MM SP cells promote Dex resistance in MP cells through exosome metastasis of SNHG16.

## 1. Introduction

Multiple myeloma (MM) is one of the most common hematological malignancies in adults worldwide [1]. Despite considerable progress being made in treatment strategies for MM, the 5-year survival rate of MM patients is less than 40%, which is mainly attributed to drug resistance and recurrence [2]. Therefore, there is an urgent need to investigate the potential drug resistance and relapse mechanisms underlying MM.

Cancer stem cells (CSCs) are a small group of tumor cells with self-renewal ability that can drive the formation and growth of tumors and may be the root source of tumor production, metastasis, recurrence, and drug resistance [3]. Side population (SP) cells, which have similar characteristics to those of CSC, have the ability to differentiate into MP cells and exhibit strong tumorigenicity [4, 5]. SP cells are also resistant to dexamethasone (Dex), a conventional chemotherapeutic agent used to treat MM [6]. However, it is vital to understand the role of SP cells in the Dex resistance of MM cells.

Exosomes are membrane-derived vesicles derived from endosomal multivesicular vesicles with a size range of 30–150 nm [7]. Studies have found that exosomes contain various bioactive molecules such as nucleic acids, proteins, and lipids, which can be transferred from donor cells to recipient cells to realize intracellular information transmission [8, 9]. Abnormal expression of long noncoding RNAs (lncRNAs) is markedly related to the Dex resistance of MM [10]. Recent studies have shown that lncRNAs such as NEAT1, CRNDE, and HOTAIR are key regulators of Dex resistance in MM [11–13]. However, whether SP cells promote Dex resistance in MP cells via exosomal lncRNAs remains unknown.

Our previous studies have found that the lncRNA SNHG16 plays a crucial role in MM proliferation [14]. In the current study, SP and MP cells were sorted from the MM RPMI-8226 cells, and the effects of SNHG16 on SP cells and MP cells on Dex resistance were investigated. Subsequently, exosomes were isolated from SP and MP cells, SNHG16 expression in exosomes was measured, coculture of exosomes and MP cells were performed, and the effects of SNHG16 on MP cell Dex resistance were investigated.

## 2. Materials and Methods

### 2.1. Cell Culture and Transfection

Human MM cells RPMI-8226 (Cell Bank of the Chinese Academy of Sciences, Shanghai, China) were cultured in RPMI-1640 (Gibco, Carlsbad, CA, USA) supplemented with 10% fetal bovine serum at 37°C in a humidified atmosphere of 5% CO_2_. Overexpression of SNHG16 (OE-SNHG16) and negative control (OE-NC) vectors, siRNAs to SNHG16 (si-SNHG16), and si-NC were purchased from GeneChem (Shanghai, China). All transfections were carried out using Lipofectamine 2000 (Invitrogen, Waltham, MA, USA) in accordance with the manufacturer's instructions.

### 2.2. SP and MP Cell Separation

SP and MP cells were sorted from the MM RPMI-8226 cells using Hoechst 33342-labeledfluorescence-activated cell sorting, as previously described [15].

### 2.3. Cell Viability Assays

Cell proliferation was evaluated using the CellTiter 96® AQueous One Solution Cell Proliferation Assay (MTS assay; Promega, Madison, WI, USA) according to the manufacturer's instructions. The cells were added to 96-well plates at concentrations of 0, 2, 5, 10, 20, 50, 100, and 200 *μ*M Dex (Sigma Aldrich) and/or incubated with 40 *μ*g exosome/well for 48 h. MTS reagent was added to the wells and incubated for 2 h. The optical density at 490 nm was measured using a microplate reader (Bio-Rad, Hercules, CA, USA). The half-maximal inhibitory concentration (IC50) and the survival rate were calculated.

### 2.4. Quantitative Real-Time PCR (qRT-PCR)

TRIzol reagent (Invitrogen) was used to extract total RNA from the cells or exosomes. The PrimeScript™ II 1st Strand cDNA Synthesis Kit (TaKaRa Bio, Dalian, China) was used to reverse transcribe the first-strand cDNA to total RNA. PCR was performed using an ABI 7500 RT-PCR system (Applied Biosystems, Foster City, CA, USA) with a SYBR® Premix Ex Taq™ Kit (TaKaRa). PCR primers were obtained from GenePharma (Shanghai, China) with the following sequences: SNHG16 forward, 5′-CCTCTAGTAGCCACGGTGTG-3′, and reverse 5′-GGCT GTGCTGATCCCATCTG-3′; aldehyde dehydrogenase 1 (ALDH1) forward, 5′-TCACAGGATCAACAGAGGTTGG-3′, and reverse 5′-GCCCTGGTGGTAGAA TACCC-3′; sex-determining region Y (SRY)-box2 (Sox2) forward, 5′-TACAGCATG ATGCAGGACCA-3′, and reverse 5′-CTCGGACTTGACCACCGAAC-3′; 18S rRNA forward, 5′-CCTGGATACCGCAGCTAGGA-3′, and reverse 5′-GCGGCGCAATACG AATGCCCC-3′; 18S rRNA served as endogenous controls for SNHG16 expression. The fold-change in the expression was computed using the 2^−ΔΔCT^ method [16].

### 2.5. Exosome Isolation, Transmission Electron Microscopy, and Nanoparticle Tracking Analysis

ExoQuick-TC precipitation solution (System Biosciences, Mountain View, CA, UAS) was used to isolate exosomes from the culture medium according to the manufacturer's instructions. A BCA kit (Beyotime, Shanghai, China) was used to measure the concentration of exosomes. To ensure the isolation of exosomes, the protein expression of TSG101 and CD63 was assessed by western blotting. Transmission electron microscopy (TEM; Tokyo, Japan) was used to identify the size and shape of the exosomes. The particle size of the exosomes was determined using nanoparticle tracking analysis (NTA; Zetaview, Particle Metrix Inc., Bavaria, Germany).

### 2.6. Western Blotting

First, total protein samples from the cells or exosomes were extracted and separated by sodium dodecyl sulfate polyacrylamide gel electrophoresis. After blocking, the membrane was incubated overnight at 4°C with diluted primary antibodies: anti-P-glycoprotein (P-gp) (ab261736, 1/1000), antimultidrug resistance-associated protein 1 (MRP1) (ab260038, 1/1000), anti-hsp70 (ab2787, 1/1000), anti-CD63 (ab134045, 1/1000), and GAPDH (ab181602, 1/10000). After incubation with the primary antibody, the PVDF membranes were rinsed and incubated with horseradish peroxidase (HRP)-labeled secondary antibody (ab205718, 1/2000) for 2 h at 25°C and then washed. Finally, the proteins were quantified using enhanced chemiluminescence (Keygentec, Nanjing, China) and a ChemiDoc™ XRS system (Bio-Rad).

### 2.7. Statistical Analysis

Data analyses were performed using SPSS 19.0 (IBM Inc., Chicago, IL, USA). All data are expressed as the mean ± standard deviation (SD), according to the data of three independent replicates. Differences between two groups were assessed using the *t*-test, while differences between more than two groups were assessed using one-way analysis of variance. Statistical significance was set at *P* < 0.05.

## 3. Results

### 3.1. SP Cells Had Remarkable Dex Resistance

**Figure 1:**
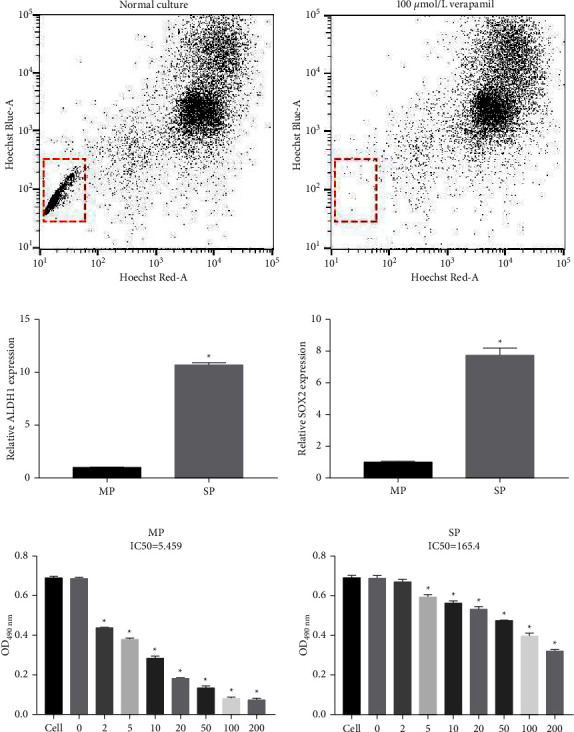
Isolation of SP and MP cells in MM cells and their Dex resistance. (a) SP cells and MP cells were isolated in RPMI-8226 cells using the Hoechst 33342 fluorescence staining method with fluorescence-activated cell sorting. (b) mRNA expression of ALDH1 and sox2 (SP markers) in SP cells and MP cells were assessed by qRT-PCR. (c) The cell viability to Dex of SP and MP cells was measured by MTS assay (^*∗*^*P* < 0.05).

To investigate the relationship between SP and MP cells in MM, SP, and MP cells were isolated from MM (Figure 1(a)). To further prove that the isolated cells were SP and MP cells, qRT-PCR was used to assess ALDH1 and Sox2 expression. ALDH1 and Sox2 mRNA expression levels were remarkably upregulated in SP cells compared with those in MP cells (Figure 1(b)), suggesting that SP and MP cells were resoundingly sorted from MM cells. To distinguish between the Dex resistance of SP and MP cells, cell viability was measured using MTS assays. The IC50 of SP cells (165.4) was remarkably higher than that of MP cells (5.454) (Figure 1(c)).

### 3.2. Isolation and Characterization of SP or MP Cell-Derived Exosomes

**Figure 2:**
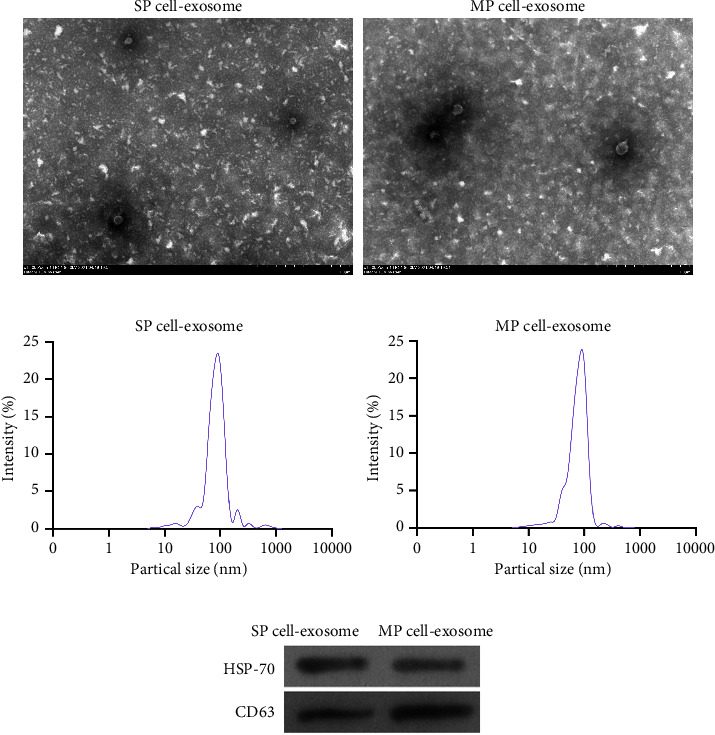
Characterization of SP or MP cell-derived exosomes. (a) Transmission electron microscopy (TEM) was applied to identify exosome size and shape. (b) Nanoparticle tracking analysis (NTA) was applied to identify exosome size. (c) The protein expression of TSG101 and CD63 was assessed using western blot.

To investigate the relationship between exosomes and SP or MP cells, the exosomes in SP and MP cells were isolated, and the identification results of TEM and NTA experiments revealed that exosomes derived from SP and MP cells had a typical dish-shapeddouble-layer membrane structure, with a diameter of 50–150 nm, suggesting that the exosomes were successfully extracted (Figures 2(a) and 2(b)). Western blotting results showed that the exosome markers HSP70 and CD63 were highly expressed in the extracted exosome samples (Figure 2(c)).

### 3.3. SP Cell-Derived Exosomes Increased Dex Resistance in MP Cells

**Figure 3:**
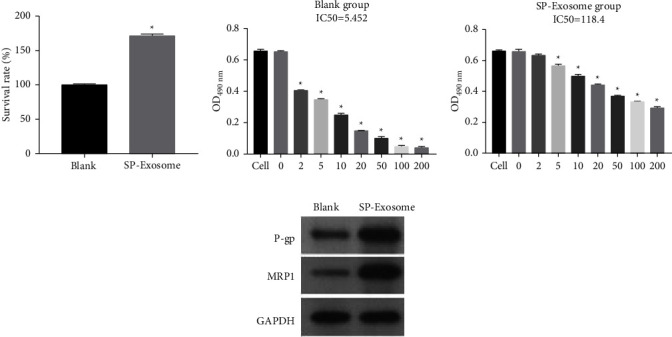
SP cell-derived exosomes induced Dex resistance in MP cells. (a) The cell proliferation of MP cells and MP cell cotreatment with Dex + SP cell-derived exosomes were assessed by MTS assay. (b and c) The cell viability to Dex of MP cells and MP cell cotreatment with Dex + SP cell-derived exosomes was measured by MTS assay. (d) The protein expression of P-gp and MRP1 in MP cells and MP cell cotreatment with Dex + SP cell-derived exosomes were detected by western blot. (^*∗*^*P* < 0.05).

To investigate the effect of SP cells on the Dex resistance of MP cells, MP cells were treated with 5 *μ*M Dex and then incubated with 40 *μ*g SP cell-derived exosomes. Compared with MP cells (blank group), the survival rate of MP cells cotreated with Dex + SP cell-derived exosomes (SP-exosome group) increased (Figure 3(a)). The Dex IC50 concentration (118.4) of SP-exosome group cells was remarkably higher than that (5.452) of the blank group cells (Figures 3(b) and 3(c)). The protein expression of the drug resistance markers P-gp and MRP1 was assessed by western blotting. P-gp and MRP1 protein expression levels were remarkably increased in SP-exosome group cells compared to those in the blank group cells (Figure 3(d)). These results suggested that MP cells acquire Dex resistance by absorbing SP cell-derived exosomes.

### 3.4. SP Cell-Derived Exosomes Could Transfer SNHG16

**Figure 4:**
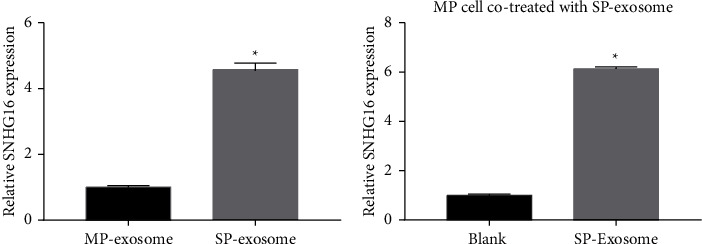
SP cell-derived exosomes could transmit the expression of SNHG16. (a) The SNHG16 expression levels in SP and MP cell-derived exosomes were measured by qRT-PCR. (b) The SNHG16 expression levels in MP cells (blank group) and MP cells incubated with SP cell-derived exosomes (SP-exosome group) were measured by qRT-PCR (^*∗*^*P* < 0.05).

SNHG16 expression in SP- and MP-derived exosomes was measured by qRT-PCR. SNHG16 expression was remarkably upregulated in SP cell-derived exosomes compared to that in MP cell-derived exosomes (Figure 4(a)). Then, SNHG16 expression in MP cells and MP cells incubated with SP cell-derived exosomes was measured. The results showed that SNHG16 expression was remarkably upregulated in MP cells incubated with SP cell-derived exosomes compared to that in MP cells (Figure 4(b)). These results suggested that SP cell-derived exosomes can transmit the expression of SNHG16 into MP cells.

### 3.5. Overexpression of SNHG16 Promoted MP Cell Dex Resistance

**Figure 5:**
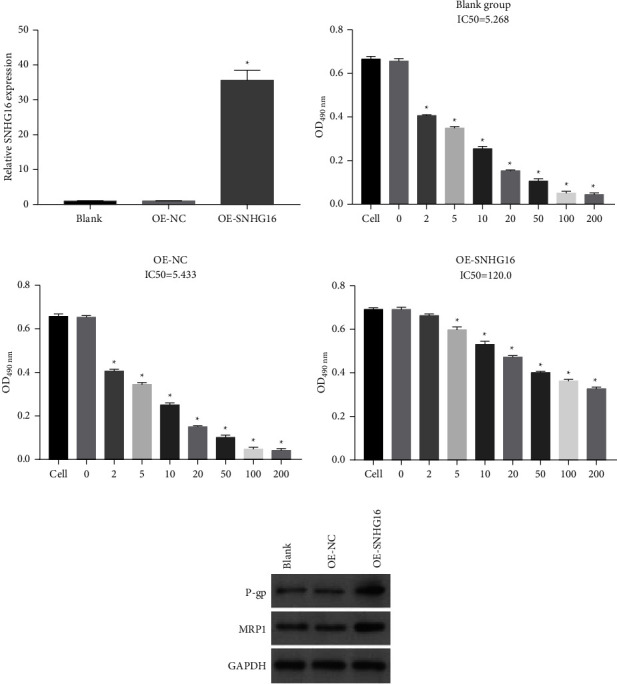
Overexpression of SNHG16 promoted MP cell Dex resistance. (a) SNHG16 expression levels in MP cells, MP cells transfected with OE-NC, and OE-SNHG16 vectors were measured by qRT-PCR. (b)–(d) The cell viability to Dex of MP cells, MP cells transfected with OE-NC, or OE-SNHG16 vectors was measured by MTS assay. (e) The P-gp and MRP1 protein expression levels in MP cells, MP cells transfected with OE-NC, or OE-SNHG16 vectors were measured by western blot (^*∗*^*P* < 0.05).

To determine the effect of SNHG16 on Dex resistance in MP cells, SNHG16 was overexpressed in MP cells by transfection with OE-SNHG16 vectors. SNHG16 expression levels in MP cells and MP cells transfected with OE-NC and OE-SNHG16 vectors were measured by qRT-PCR. The results showed that SNHG16 expression was remarkably upregulated in MP cells transfected with OE-SNHG16 vectors compared to that in MP cells and MP cells transfected with OE-NC vectors (Figure 5(a)). The cell viability to Dex assay showed that the Dex IC50 concentration of MP cells transfected with OE-SNHG16 vectors (120.0) was remarkably higher than that of MP cells (5.268) and MP cells transfected with OE-NC vectors (5.433) (Figures 5(b)–5(d)). The western blot results showed that P-gp and MRP1 protein expression levels were remarkably upregulated in MP cells transfected with OE-SNHG16 vectors compared to those in MP cells and MP cells transfected with OE-NC vectors (Figure 5(e)).

### 3.6. Silencing SNHG16 in SP Cells Hardly Affected MP Cell Dex Resistance

**Figure 6:**
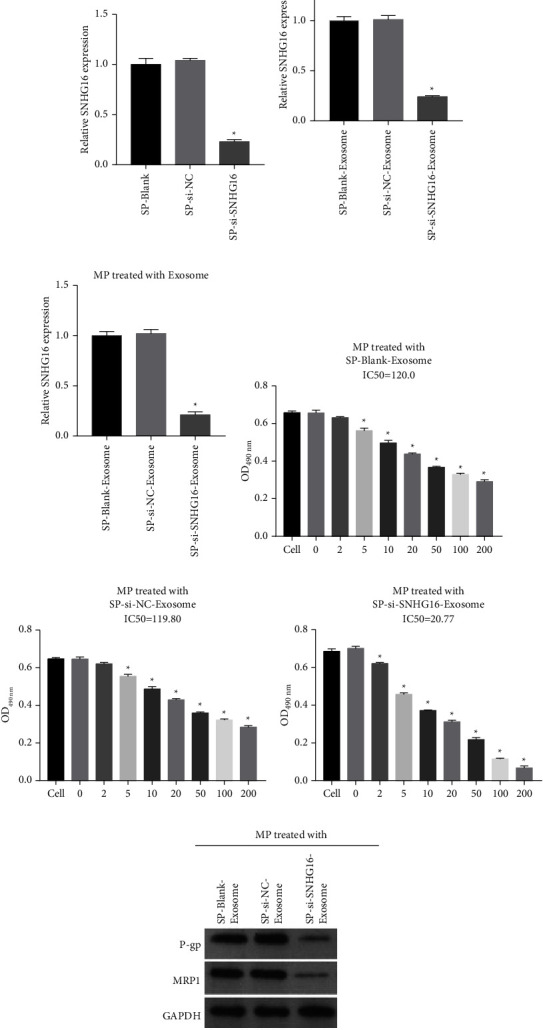
Silencing SNHG16 in SP cells hardly affected MP cell Dex resistance. (a) SNHG16 expression levels in SP cells, SP cells transfected with si-NC, and si-SNHG16 were measured by qRT-PCR. (b) The SNHG16 expression levels in SP cell exosomes, SP cells transfected with si-NC, and si-SNHG16 exosomes were measured by qRT-PCR. (c) SNHG16 expression levels in MP cells treated with exosomes. (d)–(f) The cell viability to Dex of MP cells treated with SP-blank-exosomes, SP-si-NC-exosomes, and SP-si-SNHG16-exosomes was measured by MTS assay. (g) The P-gp and MRP1 protein expression levels in MP cells treated with SP-blank-exosomes, SP-si-NC-exosomes, and SP-si-SNHG16-exosomes were measured by western blot (^*∗*^*P* < 0.05).

To demonstrate whether MP cells conferred Dex resistance via incorporation into SNHG16 in SP cell-derived exosomes, the expression of SNHG16 in SP cells was knocked down by transfection with si-SNHG16 (Figure 6(a)). Consistently, the expression of SNHG16 in SP cell-derived exosomes was also knocked down (Figure 6(b)). MP cells were then cocultured with SP-si-SNHG16-exosomes, and SNHG16 expression was remarkably downregulated in MP cells treated with SP-si-SNHG16-exosomes compared with that in MP cells treated with SP-si-NC-exosomes and SP-blank-exosomes (Figure 6(c)). Moreover, the IC50 concentration of Dex in MP cells treated with SP-si-SNHG16-exosomes (20.77) was remarkably lower than that in SP cells treated with SP-si-NC-exosomes (119.8) and SP-blank-exosomes (120.0) (Figures 6(d)–6(f)). The western blot results showed that P-gp and MRP1 protein expression levels were remarkably downregulated in MP cells treated with SP-si-SNHG16-exosomes compared to those in SP cells treated with SP-si-NC-exosomes and SP-blank-exosomes (Figure 6(g)). These results suggest that MP cells could acquire drug resistance by absorbing SNHG16 in SP cell-derived exosomes.

## 4. Discussion

MM is still considered incurable and seriously threatens the health of people. Dex is the most conventional chemotherapeutic drug used for the treatment of MM, and its innate or achieved drug resistance is widely associated with a poor prognosis in MM [17]. The mechanisms of Dex resistance in MM have been studied previously [18]. However, the mechanism by which they acquire resistance remains unclear. In this study, we successfully isolated SP and MP cells from MM cells. In addition, we found that SP cells were more resistant to Dex than to MP cells. This is consistent with previous studies [19]. Exosomes mediate intercellular communication by transferring information from donors to target cells [20]. Tumor cells and tumor-associated stromal cells can release and receive exosomes and are widely involved in MM progression [21]. In this study, exosomes were successfully isolated from SP and MP cells. Moreover, the survival rate and Dex resistance of MP cells cotreated with Dex + SP cell-derived exosomes were enhanced, suggesting that MP cells could acquire Dex resistance by absorbing SP cell-derived exosomes.

Recently, increasing evidence has demonstrated that exosomes serve as a medium for information exchange between different cell types through the transmission of constituents [22]. The effects of exosomal lncRNAs on drug resistance have also been previously demonstrated. Exosomal H19 promotes Dex resistance in breast cancer, and exosomal SNHG7 promotes docetaxel resistance in lung adenocarcinoma [23, 24]. However, the functions of exosomal lncRNAs in MM remain unclear. To elucidate the functional mechanism and resistance to Dex in MM, we focused on lncRNAs, which have been demonstrated to play a vital role in cancer chemoresistance [25]. SNHG16 has oncogenic effects [26]. In our previous study, SNHG16 was upregulated in MM and promoted MM cell proliferation by sponging miR-342-3p [14]. Here, SNHG16 expression was remarkably enhanced in SP cell-derived exosomes compared to MP cell-derived exosomes. In addition, SNHG16 was transferred from SP cells to MP cells, which was first found in exosomes. Some studies have shown that SNHG16 contributes to chemotherapy resistance in cancer. For example, knockdown of SNHG16 inhibited cell function and sorafenib resistance in Hep3B and HepG2 cell lines [27], and SNHG16 silencing weakened cisplatin resistance in neuroblastoma cells [28]. The detailed mechanisms of SNHG16 in MM have not yet been elucidated. Here, overexpression of SNHG16 remarkably enhanced Dex resistance in MP cells. However, when si-SNHG16 downregulated the expression of SNHG16 in SP cell-derived exosomes, SNHG16 expression and Dex resistance were not remarkably enhanced in MP cells treated with SP-si-SNHG16-exosomes. These findings indicate that silencing of SNHG16 in SP cell-derived exosomes prevented MP cells from acquiring SNHG16 and thus failed to enhance Dex resistance.

This study has three main limitations. First, the regulatory mechanism of exosomal SNHG16 in MP remains unclear, the mechanism by which SNHG16 in SP cells is secreted into exosomes also remains unclear, and lastly the role of exosome-derived SNHG16 must be confirmed by *in vivo* experiments.

**Figure 7:**
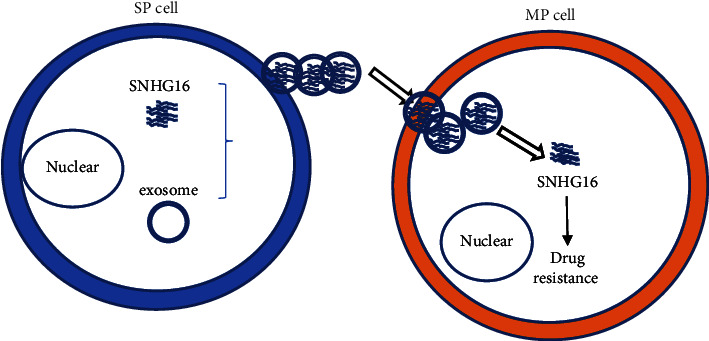
MM SP cells promote Dex resistance of MP cells through exosome metastasis of SNHG16.

Taken together, the present findings suggest that MM SP cells promote Dex resistance in MP cells through exosome metastasis of SNHG16 (Figure 7). The functional role of lncRNAs in SP cell-derived exosomes will help discover new and more efficient strategies to reverse drug resistance.

## Data Availability

The data used to support the findings of this study are included within the article.
